# Safe management of surgical smoke in the age of COVID-19

**DOI:** 10.1002/bjs.11679

**Published:** 2020-05-03

**Authors:** N G Mowbray, J Ansell, J Horwood, J Cornish, P Rizkallah, A Parker, P Wall, A Spinelli, J Torkington

**Affiliations:** Department of General Surgery, University Hospital of Wales, Cardiff, UK; School of Medicine, Cardiff University, Cardiff, UK; Isca Healthcare Research, Caerleon, UK; Department of General and Minimally Invasive Surgery, Istituto Clinico Humanitas, Rozzano, Italy

## Abstract

**Background:**

The COVID-19 global pandemic has resulted in a plethora of guidance and opinion from surgical societies. A controversial area concerns the safety of surgically created smoke and the perceived potential higher risk in laparoscopic surgery.

**Methods:**

The limited published evidence was analysed in combination with expert opinion. A review was undertaken of the novel coronavirus with regards to its hazards within surgical smoke and the procedures that could mitigate the potential risks to healthcare staff.

**Results:**

Using existing knowledge of surgical smoke, a theoretical risk of virus transmission exists. Best practice should consider the operating room set-up, patient movement and operating theatre equipment when producing a COVID-19 operating protocol. The choice of energy device can affect the smoke produced, and surgeons should manage the pneumoperitoneum meticulously during laparoscopic surgery. Devices to remove surgical smoke, including extractors, filters and non-filter devices, are discussed in detail.

**Conclusion:**

There is not enough evidence to quantify the risks of COVID-19 transmission in surgical smoke. However, steps can be undertaken to manage the potential hazards. The advantages of minimally invasive surgery may not need to be sacrificed in the current crisis.

## Graphical Abstract

The COVID-19 pandemic is unprecedented and raises concerns regarding transmission of the virus within surgical smoke. This review summarizes the current evidence and provides advice for its management.

**Graphical Abstract:**
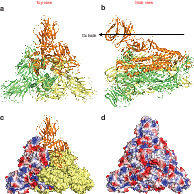

Bad for your health?

## Introduction

The 2020 pandemic of the novel coronavirus, COVID-19, has raised concerns about the risk of virus transmission to staff in the operating room. This relates not just to intubation and extubation of the airway during anaesthesia, but also to the release of potential infectious particles in laparoscopic smoke or plume.

The risks of laparoscopic smoke, or plume, have been recognized for a long time, but the advent of COVID-19 has brought its importance into sharp relief^1^. The Society of American Gastroenterology and Endoscopic Surgeons updated their advice on 30 March 2020^2^: ‘Although previous research has shown that laparoscopy can lead to aerosolization of blood-borne viruses, there is no evidence to indicate that this effect is seen with COVID-19, nor that it would be isolated to MIS [minimally invasive surgery] procedures. Nevertheless, erring on the side of safety would warrant treating the coronavirus as exhibiting similar aerosolization properties. For MIS procedures, use of devices to filter released CO_2_ for aerosolized particles should be strongly considered’.

A rapid, joint publication from Italian and Chinese surgeons^3^ has shared excellent advice based on their experiences in the preceding months. The UK and Ireland Intercollegiate Board has also continued to update its guidance. It moved from a statement that ‘laparoscopy should generally not be used’ to its most recent guideline on 27 March 2020^4^, which states: ‘Laparoscopy is considered to carry some risks of aerosol-type formation and infection and considerable caution is advised. The level of risk has not been clearly defined and it is likely that the level of PPE [personal protective equipment] deployed may be important. Advocated safety mechanisms (filters, traps, careful deflating) are difficult to implement. Consider laparoscopy *only* in selected individual cases where clinical benefit to the patient substantially exceeds the risk of potential viral transmission in that particular situation’. This expeditious advice is a pragmatic response to the widespread anxiety surrounding possible virus transmission in the operating room.

This article reviews the best available evidence to understand the risk of transmission of COVID-19 in laparoscopic smoke, and what steps, based on physical properties of the virus, may be best placed to reduce this and justify continuing laparoscopic surgery under strict safety guidelines.

## Risk of virus transmission in surgical smoke

There is currently no evidence that COVID-19 is transmissible through surgical smoke. Previous studies have, however, demonstrated the presence of different viruses in surgical smoke, including: corynebacterium, human papillomavirus (HPV), poliovirus, human immunodeficiency virus (HIV) and hepatitis B^5–8^. The aerosol produced by laparoscopic or robotic surgery, particularly when using low-temperature ultrasonic devices, may not effectively deactivate the cellular components of a virus^3^.

The possibility of disease transmission through surgical smoke does exist in humans, but documented cases are rare^9^. HPV transmission during anogenital surgery is the most widely reported in the literature. This is most likely due to the direct contact of electrocautery on an infected field. Liu and colleagues^9^ outlined four articles^10–13^ linking surgical smoke to the transmission of HPV (types 6, 11 and 13), progressing to oropharyngeal squamous cell carcinoma. These cases occurred in otherwise fit healthcare professionals performing gynaecological surgery with no other risk factors for the disease. Another study^14^ found that one in five surgeons, and three in five nurses, tested positive for HPV after performing operations for laryngeal and urethral papillomas^14^. The HPV genotypes in the infected healthcare professional were identical to those identified in the patient.

In addition to HPV transmission, Baggish and co-workers reported that HIV proviral DNA was captured in the inner lumen of smoke evacuation tubing after *in vitro* laser vaporization of cultured HIV cells^15^. Kwak *et al*.^16^ further revealed that hepatitis B virus (HBV) was present in surgical smoke^16^. In this study, surgical smoke was collected during laparoscopic or robotic surgery in 11 patients. HBV was detected in ten of the 11 samples of surgical smoke, including that from non-hepatic surgery. This suggests that bloodborne viruses may be present within surgical smoke. Although the transmission of COVID-19 is, at present, felt to occur primarily through respiratory droplets, there remains a theoretical risk of virus aerosolization during minimal access surgery^17,18^.

## Physical properties of the coronavirus

It is essential to understand the physical properties of the virus in order to predict the risk of transmission and the effectiveness of preventative measures. The correct term for the virus causing COVID-19 is *Sarbecovirus* severe acute respiratory syndrome coronavirus 2 (SARS-CoV2), which is a member of the betacoronavirus family (subgenus *Sarbecovirus*). Like its close relatives, Middle East respiratory syndrome coronavirus (MERS-CoV) and SARS-CoV, it is believed to have its origins in bats. Sequence analysis from cases around the world strongly indicate that the pandemic resulted from a single recent emergence of the virus from an animal reservoir.

SARS-CoV2 is a positive-sense single-stranded RNA virus, containing a linear genome of approximately 30 kb. Like other members of the coronavirus family, the virion is typically around 120 nm in diameter, although this can vary from 50 to 200 nm^19^. As an enveloped virus, the RNA genome is contained within a lipid bilayer containing several proteins: the N protein binds and stabilizes the RNA genome, and the envelope (E), membrane (M) and spike (S) proteins make up the envelope. Like other members of the SARS family, the virus uses the heavily glycosylated, extended spike protein to mediate initial cellular engagement and begin the process of infection^20^. The S protein of SARS-CoV2 engages the angiotensin-converting enzyme 2 receptor (ACE2) as a high-affinity primary attachment receptor^21^. ACE2 expression is abundant on human airway epithelial cells, consistent with efficient transmission occurring via respiratory droplets. Other co-receptors may also be involved in cellular infection, including CD147 (basigin, BSG) and CD26^20,22^. In each case, the interaction is thought to occur with the S protein, which appears pivotal for cellular tropism.

**Fig. 1:**
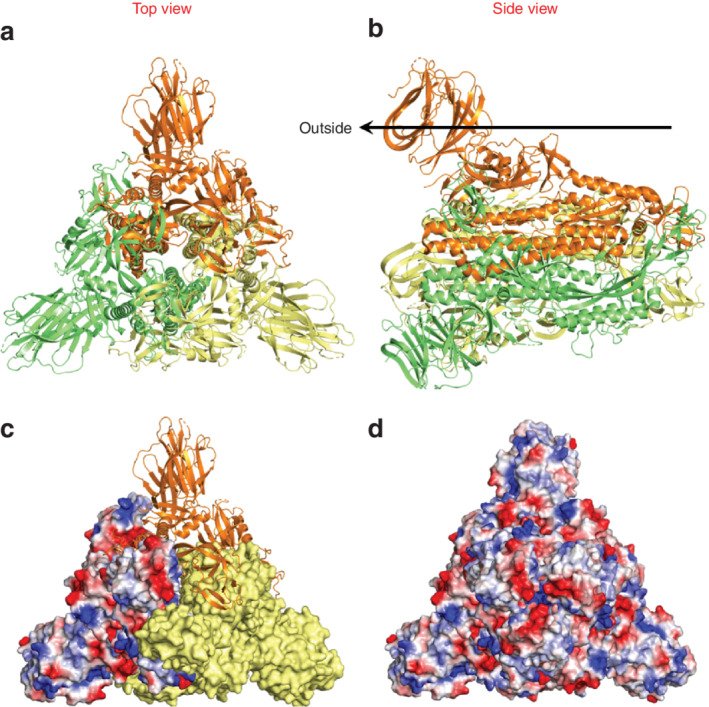
Structure and isoelectric properties of SARS-CoV2 spike protein

The extended S protein is a trimeric protein, each monomer being approximately 1100 amino acids in length. The structure of the protein (based on Protein Data Bank (PDB) 6CRV) is depicted in *Fig. 1a,b*. Binding of ACE2 to S protein occurs at the apical domain. Although the overall net virion charge has not yet been evaluated, it is possible to assess the surface electric potential of individual capsid proteins where structures have been elucidated, as depicted for the S protein in *Fig. 1c,d*. *Fig. 1d* shows the full surface electric potential of the spike protein, as viewed towards the virion. Areas of both positive and negative charge are visible in approximately equal abundance, although the central depression, where ACE2 is thought to bind, and one of the few regions of the S protein that is devoid of glycosylation, is seen to be relatively negatively charged.

Structure of the SARS-CoV2 spike protein as viewed from top down (**a**) and in cross-section (**b**), based on Protein Data Bank (PDB) entry 6CRV. The trimeric assembly, as viewed from top down, is shown as an amalgam cartoon (top), solid surface (right, yellow) and electric potential (left) (**c**), and the full trimer overlaid with the full surface electric potential (**d**), highlighting a trefoil of negative charge (red) around the central point.

## Operating theatre set-up

Excellent theatre discipline and the use of checklists is highly recommended to minimize the risk of surgery and anaesthesia, regardless of surgical approach. Expert recommendations from China suggest using a designated operating theatre for COVID-19-positive patients^23^. This allows patient movements to be planned, limiting the contamination of other clinical areas. The flow of theatre staff can also be mapped to create designated areas for donning and doffing full personal protective equipment. The minimum number of staff should be present in the operating theatre, with the minimum amount of equipment. A runner can acquire further stock as required, but should be mindful to minimize the number of entries into the operating theatre.

In deciding which operating theatre to use, the theatre ventilation system should be considered. Negative pressure ventilation can limit the contamination of surgical smoke into neighbouring areas through doors and vents. During the SARS outbreak in Hong Kong in 2004, Chow and colleagues^24^ described how they converted existing systems into negative pressure systems. This may not always be feasible, and so the recommendations from the UK and Ireland surgical colleges are that positive pressure ventilation should be halted during the procedure and for at least 20 min after the patient has left theatre^4^. The risks of positive pressure ventilation, however, have not been quantified. If the virus is present in laparoscopic smoke, it must make its way to the edge of the theatre and then must have an uplift in theatre corridors at a concentration sufficient to be infective. Any virus load is likely to be diluted massively by the time this happens.

Unidirectional air flow (laminar flow) systems remain controversial in their ability to decrease surgical-site infections and so are not recommended by the WHO^25,26^. They produce a continuous flow of highly filtered ultraclean air and can display 99·97 per cent efficiency in removing airborne particles of 0·3 μm and larger^27^. This would imply an effectiveness in clearing COVID-19, but it is also necessary to consider having multiple theatres available as sufficient downtime between procedures is essential to ensure full decontamination. If smoke is a risk factor, in a laminar flow theatre it will be pushed to the floor, away from staff, and the inhalation risk diminished.

There is a limited amount of evidence related to the choice of tissue-cutting energy device used. Monopolar electrocautery using Teflon® (Chemours, Delaware, USA) blades and a feedback mechanism to automatically reduce the voltage produces less surgical smoke^28^. The main laparoscopic alternatives to electrocautery are the ultrasonic energy devices such as Harmonic Scalpel (ACE®; Ethicon Endo-Surgery, Cincinnati, Ohio, USA). Although such devices can add efficacy and speed to some laparoscopic procedures, more viable cells have been detected in the smoke from ultrasonic energy devices^29^.

## Mechanical measures for smoke evacuation

The operating room ventilation system is not the only method of smoke extraction; protection from surgical smoke can also be achieved by local extraction at the site of surgery or by the use of personal filtration masks. There are several benefits to using smoke extractors; they keep the surgical field clear, prevent corrosion of equipment due to released chemical, and reduce odour. However, for the purpose of this review, the main benefit is that they capture the smoke close to the source of emission, therefore minimizing exposure of healthcare professionals to potential contaminants and maintaining a safer environment. For this reason, the surgical colleges in the UK currently recommend their use^4^.

The particulate removal capability of smoke evacuator devices is, by design, limited to the efficiency and size of their filters. Other factors to consider include: the minimum flow rate (at least 0·012 m^3^/s), the ability to vary both the flow rate and noise level (ideally below 60 dB), ergonomic features, portability, cost-effectiveness and ease of maintenance^30^.

There are a number of types of filter available. Charcoal filters use activated charcoal; they can absorb both gas and vapour, and can eliminate strong-smelling gases. Coconut shell charcoal is better at absorbing particulate matter than wood-based charcoal owing to its greater internal pore area^31^. High-efficiency particulate air filters act to filter suspended compounds. They can retain particles larger than 0·3 μm at an efficiency rate of 99·97 per cent^32^. Ultralow particulate air (ULPA) filters retain 99·9 per cent of particles at 0·1 μm and are a depth filter, filtering matter by different methods depending on the particle size^32^. This makes them ideal for removing the particles created in electrosurgical and laser procedures.

The Association of periOperative Registered Nurses guidelines^33^ state that perioperative personnel should use ULPA filters routinely for surgical smoke. Currently the most effective smoke evacuation system is the triple-filter system, which includes a prefilter that captures large particles, a ULPA filter, and a special charcoal that captures the toxic chemicals found in smoke. Triple-filter systems often have variable suction volume capacity to accommodate various levels of smoke production.

Most commonly these filters are used in portable evacuation machines, but in some instances a filter can be used in the wall suction system to safely capture small amounts of smoke. Depending on the environment, these filters may require replacement after each use or each day. Most of the evacuation devices have an inbuilt alarm or an indicator light to signal a required change. A change of filter is mandated when the suction pressure decreases or there is a lingering odour in the air. The contaminated filter may be considered as infectious or regulated waste depending on the waste disposal protocol of the facility^34^. *Table 1* summarizes the currently available smoke evacuation devices.

**Table 1:** Summary of commercially available smoke evacuation systems

	CONMED	CooperSurgical®	Ethicon	Medtronic	Olympus	Stryker	Northgate
Product name	AirSeal® (laparoscopic) PlumePen® (open) Buffalo Filter® Smoke Management	SeeClear Plume-away	Megadyne™ MegaVac PLUS™ MegaVac™ MiniVac™	ValleyLab RapidVac™	UHI-4	Pneumoclear™ PureView™ Neptune™ (open) SafeAir™ (open) Photonblade™ (open) Smoke Evac Retractors™ (open)	Nebulae™ I system
Open	Yes	No	Yes	Yes	No	Yes	No
Laparoscopic	Yes	Yes	MegaVac PLUS™ only	Yes	Yes	Yes	Yes
ULPA	Yes	Yes	Yes	Yes	No	Yes	Yes
Micron filtration	0·012	0·1	0·1	0·1–0·2	n.a.	0·051–0·1	0·12
Passive or active evacuation	Active	Passive	Active	Active	Active	Active	Active

Reproduced from a document published by the Society of Gastrointestinal and Endoscopic Surgeons in conjunction with their guidelines for surgeons concerning the use of laparoscopy during the current COVID-19 pandemic^2,35^. ULPA, ultralow particulate air; n.a., not available. CONMED, Utica, New York, USA; CooperSurgical®, Trumbull, Connecticut, USA; Ethicon, Somerville, New Jersey, USA; Medtronic, Dublin, Ireland; Olympus, Shinjuku City, Tokyo, Japan; Stryker, Kalamazoo, Michigan, USA; Northgate, Elgin, Illinois, USA.

Choi and colleagues^36^ assessed surgical smoke from 20 patients undergoing transperitoneal laparoscopic nephrectomy for renal cell carcinoma. Surgical smoke (prefilter) was collected 20 min after the electrocautery device was first used during the procedure and 2 h after the filter had been applied. The sample was analysed by gas chromatography–mass spectroscopy. Strong carcinogens were eliminated by more than 85 per cent by using the activated carbon fibre filter. Seipp and co-workers^37^ tested the efficiency of portable smoke evacuation systems and found that filtration reduced surgical smoke by up to 99 per cent. However, this was accompanied by high noise levels that exceeded recommended limits.

Despite recommendations from various professional organizations advocating the use of smoke extraction devices in operating rooms, these measures are not being used widely because of excessive noise, high cost, equipment maintenance issues, large bulky devices, and resistance or complacency from clinical staff^38–40^.

## Laparoscopic trocars and release of pneumoperitoneum

All minimally invasive surgeons will be aware that the trocar represents a point of weakness, by penetrating the abdominal wall and allowing the inadvertent escape of pressurized carbon dioxide from the pneumoperitoneum. This is an obvious area of concern in a COVID-19-positive patient, and remains a risk throughout the procedure as displacement of the port is not uncommon.

Pressurized intraperitoneal aerosol chemotherapy (PIPAC) delivers aerosol chemotherapy under pressure during laparoscopy for the treatment of peritoneal carcinomatosis. By its very nature, the procedure has necessitated a high level of investigation into ways of mitigating the risk of gas leakage during surgery. Both experimental and human models have shown that, with the development of standard operating procedures (SOPs) for laparoscopy, the risk of contamination of the operating theatre environment with potentially harmful chemotherapy agents is negligible^41^. The authors propose that these procedures could be applied to minimally invasive surgery on COVID-positive patients, similarly negating the risk of virus escape during surgery.

Open Hasson umbilical port insertion may result in a larger defect in the fascia than is required for the port, allowing pressurized carbon dioxide to escape around the trocar. PIPAC surgeons frequently use either off-midline open cutdown or alternative first entry techniques (Veress, optical insertion), which result in a fascia defect that is more snug around the port, reducing the risk of gas leakage at this site.

PIPAC SOPs recommend the use of balloon ports. Balloon-secured trocars reduce the chance of inadvertent displacement of the trocar during instrument changes, thus reducing the risk of loss of pneumoperitoneum to the operating theatre environment. Trocars also contain a valve preventing gas leakage when an instrument is passed through into the peritoneum; surgeons should be aware of the inherent risks if the valve should become damaged. In addition, the authors believe that, when the balloon is inflated inside the peritoneum and traction is applied against the posterior rectus sheath, a more secure seal is achieved around the body of the trocar, therefore reducing the risk of gas leakage.

Ten-millimetre ports should be closed under direct vision once the pneumoperitoneum has been safely evacuated using techniques described elsewhere in this article. The authors recommend against the use of devices such as EndoClose™ (Medtronic, Dublin, Ireland), which require maintenance of the pneumoperitoneum to facilitate closure, as this will increase the risk of gas leakage.

Pneumoperitoneum should be maintained throughout the procedure at the lowest possible pressure. At the end of the procedure, the pneumoperitoneum should be removed in a controlled fashion by first switching off the insufflation, and then aspirating the pneumoperitoneum using a suction device (preferably passed through a filter mechanism)^42^. If specimen extraction is required, the pneumoperitoneum should be decompressed safely as above before making an incision for specimen extraction.

## Non-filtration devices

The Ultravision™ system (Alesi Surgical, Cardiff, UK) removes smoke and particulate matter produced during electrosurgical procedures, as an aid to maintaining a clear visual field. Active throughout surgery, the system prevents the build-up of smoke by electrostatically precipitating particulate matter generated within the peritoneal cavity during the use of electrosurgical tools. It has been shown to negate the periprocedural venting of insufflation gas to facilitate clearance of smoke^43^.

The principle on which the Ultravision™ system operates is derived from electrostatic precipitators that are widely used to remove fine particulate matter from exhaust gases produced during, for example, the commercial manufacture of cement and paper. The system comprises a generator and a single-use electrode that together create negative ions in the abdominal cavity. These ions impart a transient negative charge to the particles of surgical smoke as they are created. The electrostatically charged particles are attracted to the patient tissue owing to the presence of the standard patient return electrode used during surgery. The charge is neutralized as each particle precipitates on to the surface of the peritoneal wall, where it remains unchanged physically and chemically^44,45^. Ultravision™ has also been used to enhance safety in PIPAC^46^. The functionality of the system is not restricted by particle size, and it has been demonstrated to remove over 99 per cent of all smoke particulates larger than 7 nm in size^47^.

## Conclusion

There is not enough evidence available to allow a balanced risk estimation for the infectious nature of surgical smoke. It is unclear whether laparoscopic smoke represents a greater risk than that created during open surgery. Given the novel status of the COVID-19 pathogen, and the evidence of other viruses being present in surgical smoke, maximizing intraoperative precautions at this time would seem prudent. There has never been a time when knowledge and guidelines are changing on an almost daily basis as a result of the unprecedented worldwide communication and information sharing. Minimal access surgery offers advantages to patients in terms of hospital stay. With strict locally agreed theatre protocols aligned to evidence-based protective measures, it may be possible to continue to offer minimal access surgery to those who might benefit most.

## Disclosure

J.T. holds stock in Alesi Surgical and has received educational grants from Medtronic and Ethicon. The authors declare no other conflict of interest.
